# Supplementary material for: Characterization of Two Mouse Chd7 Heterozygous Loss-of-Function Models Shows Dysgenesis of the Corpus Callosum and Previously Unreported Features of CHARGE Syndrome
Source: Int J Mol Sci. 2022 Sep 29;23(19):11509. doi: 10.3390/ijms231911509 (PMC9569499; doi:10.3390/ijms231911509)
Supplement: Supplementary file 1 [file ijms-23-11509-s001.zip › ijms-1862038-supplementary.pdf]

Supplementary Materials

Supplementary Table S1: Modified SHIRPA (SmithKline Beecham, Harwell, Imperial College, Royal London Hospital, phenotype assessment) in 7 animals of each genotype for *Chd7*$^{+/tm2a}$ and 12 wild-type and 11 mutants for the *Chd7*$^{+/Whi}$ model.

Chd7^{tm2a}

Dysmorphology	*normal* *Chd7*$^{+/+}$	*abnormal* *Chd7*$^{+/+}$	*normal* *Chd7*$^{+/tm2a}$	*abnormal* *Chd7*$^{+/tm2a}$
Skin Pigmentation	6	1	3	4
Skin Condition	7	0	7	0
Coat Colouration	7	0	7	0
Coat Texture	3	4	4	3
Head Size	7	0	7	0
Head Morphology	7	0	7	0
Snout Morphology	7	0	7	0
Mouth Morphology	7	0	7	0
Incisor	7	0	7	0
Genitalia	7	0	7	0
Genitalia Morphology	7	0	7	0
Genitalia Size	7	0	7	0
Forelimb Size	7	0	7	0
Forepaw Size	7	0	7	0
Forepaw Foot Pads	7	0	7	0
Forepaw Nails	7	0	7	0
Hindlimb Size	7	0	7	0
Coat Intact	7	0	7	0
Incisor Colour	7	0	7	0
Ear Morphology	7	0	7	0
Ear Size	7	0	7	0
Eyes	7	0	7	0
Eye Colouration	7	0	7	0
Eye Size	7	0	7	0
Whiskers	6	1	4	3
Hind Paw Size	7	0	7	0
Hindlimb Foot Pads	7	0	7	0
Hindlimb Nails	7	0	7	0
Tail Morphology	7	0	7	0
Tail Length	7	0	7	0

Chd7^{Whi}

Dysmorphology	*normal Chd7*$^{+/+}$	*abnormal Chd7*$^{+/+}$	*normal Chd7*$^{+/Whi}$	*abnormal Chd7*$^{+/Whi}$
Skin Pigmentation	12	0	11	0
Skin Condition	11	1	10	1
Coat Colouration	6	6	3	8
Coat Texture	8	4	2	9
Head Size	12	0	11	0
Head Morphology	3	9	1	10
Snout Morphology	12	0	11	0
Mouth Morphology	12	0	11	0
Incisor	12	0	11	0
Genitalia	12	0	11	0
Genitalia Morphology	12	0	11	0
Genitalia Size	10	2	10	1
Forelimb Size	12	0	11	0
Forepaw Size	12	0	11	0
Forepaw Foot Pads	12	0	11	0
Forepaw Nails	12	0	10	1
Hindlimb Size	12	0	11	0
Coat Intact	12	0	11	0
Incisor Colour	12	0	11	0
Ear Morphology	12	0	11	0
Ear Size	12	0	11	0
Eyes	12	0	11	0
Eye Colouration	12	0	9	2
Eye Size	8	4	5	6
Whiskers	12	0	11	0
Hind Paw Size	11	1	11	0
Hindlimb Foot Pads	12	0	11	0
Hindlimb Nails	9	3	10	1
Tail Morphology	12	0	11	0
Tail Length	12	0	11	0

Supplementary Table S2: Body composition in *Chd7*$^{+/Whi}$ (4 mutants versus 5 wild type) and in *Chd7*$^{+/tm2a}$ mice (9 mutants versus 11 wild-type) raw data are given together with percent increase or decrease and t-test statistics.

Male Lean Mass (g)		Male Fat Mass (g)		Male Fat Percentage Estimate (%)		Male Body Length (cm)		Male Bone Mineral Density (mg/cm^2)		Male Bone Mineral Content (g)	
Chd7+/+	*Chd7+/Whi*	*Chd7+/+*	*Chd7+/Whi*	*Chd7+/+*	*Chd7+/Whi*	*Chd7+/+*	*Chd7+/Whi*	*Chd7+/+*	*Chd7+/Whi*	*Chd7+/+*	*Chd7+/Whi*
28.15	21.59	16.77	3.09	37.34	12.54	10.9	9.7	0.056	0.0526	0.5688	0.5198
29.92	23.98	14.86	17.97	33.19	42.83	10.8	10.6	0.0583	0.0573	0.6529	0.587
28.36	20.83	16.21	2.87	36.36	12.11	10.4	9.8	0.0575	0.0566	0.6286	0.5276
27.63	25.4	15.87	17.99	36.48	41.46	11.1	10.2	0.0585	0.0571	0.5887	0.6166
30.23	21.18	14.59	3.44	32.56	13.97	11.6	9.7	0.0632	0.057	0.6875	0.5884
30.55	24.62	18.2	18.72	37.34	43.2	11.4	10.1	0.0584	0.0555	0.6362	0.5967
28.06	22.64	15.37	4.68	35.38	17.13	10.9	10.2	0.0586	0.06	0.6297	0.6126
26.12	30.34	17.68	16.25	40.37	34.88	10.2	10.9	0.0575	0.0588	0.5979	0.6413
29.16	25.01	15.66	3.6	34.94	12.6	11.2	10.1	0.0589	0.0553	0.5542	0.5928
27.17		12.71		31.86		11		0.0611		0.6706	
29.43		16.89		36.46		11.3		0.0582		0.5666	
Percent increase (+) or decrease (-)	-16.2911		-38.0464		-28.1148		-7.62509		-3.50081		-4.79149
T.test	0.04005		0.045146		0.030727		0.140299		0.303475		0.28142
Chd7+/+	*Chd7+/tm2a*	*Chd7+/+*	*Chd7+/tm2a*	*Chd7+/+*	*Chd7+/tm2a*	*Chd7+/+*	*Chd7+/tm2a*	*Chd7+/+*	*Chd7+/tm2a*	*Chd7+/+*	*Chd7+/tm2a*
25.14	19.15	5.8	12.25	18.76	39	11.4	10	0.0566	0.0498	0.6051	0.4694
23.88	19.97	17.86	7.22	42.79	26.54	10.2	9.8	0.0486	0.0524	0.4721	0.489
21.01	21.37	17.61	5.74	45.6	21.17	10.9	10.2	0.0501	0.0523	0.5259	0.5066
23.66	19.31	19.97	12.52	45.77	39.34	10.5	9.7	0.055	0.0491	0.6352	0.4741
24.95		6.45		20.53		10.5	9.6	0.0579		0.6032	
						10.7	10.1				
						10.2					
Percent increase or decrease	-15.9221		-30.3257		-9.1597		-6.85484		-5.10813		-14.6973
T.test	0.005278		0.313898		0.705167		0.003311		0.253216		0.047928

Supplementary Table S3: Hot plate test and raw data for latency to first response in seconds in 7 animals of each genotype for *Chd7*$^{+/tm2a}$ and 12 wild-type and 11 mutants for the *Chd7*$^{+/Whi}$ model. T-test statistics given in main text.

Chd7+/+	*Chd7+/tm2a*	*Chd7+/+*	*Chd7+/Whi*
6.5	7.3	9.1	16
7.7	7.6	11.9	25
17.5	14.1	15.3	22.3
12.1	5.8	13.6	22.3
11.7	21.7	7.7	16.6
7.3	14.8	14.1	10.4
7.3	6.1	14	30
		19.9	11.4
		10.6	8.8
		17.5	15.6
		10.1	30
		10.3	

Supplementary Table S4: X-ray screen in 14 weeks-old animals in 6 animals of each genotype for *Chd7*$^{+/tm2a}$ and 11 wild-type and 9 mutants for the *Chd7*$^{+/Whi}$ model.

Chd7^{Whi}

Dysmorphology	normal Chd7$^{+/+}$	abnormal Chd7$^{+/+}$	normal Chd7$^{+/tm2a}$	abnormal Chd7$^{+/tm2a}$
Skull shape	5	6	5	4
Zygomatic bone	11	0	9	0
Maxilla	11	0	9	0
Scapula	7	4	7	2
Clavicle	11	0	9	0
Normal Number of Ribs (Right)	11	0	9	0
Normal Number of Ribs (Left)	11	0	9	0
Shape of ribs	11	0	9	0
Shape of spine	11	0	9	0
Scoliosis	4 (no)	3 (yes)	4 (no)	2 (yes)
Normal Number of cervical vertebrae	11	0	9	0
Humerus	11	0	9	0
Radius	11	0	9	0
Ulna	11	0	9	0
Femur	11	0	9	0
Tibia	11	0	9	0
Shape of Ribcage	11	0	9	0
Fusion of Ribs	11	0	9	0
Pelvis	11	0	9	0
Normal number of Thoracic vertebrae	11	0	9	0
Normal number of Lumbar vertebrae	11	0	9	0
Normal number of Pelvic vertebrae	11	0	3	6 (5 with 1 extra)
Normal number of Caudal vertebrae	11	0	9	0
Transition vertebrae	7 (no)	4 (yes)	3 (no)	6 (yes)
Shape of vertebrae	11	0	7	2
Fusion of vertebrae	3 (yes)	8 (no)	5 (yes)	4 (no)
Processes on vertebrae	8	3	6	3
Transverse Processes	6	1	5	1
Fusion Processes	7	0	6	0
Fibula	11	0	9	0
Normal number of digits	11	0	9	0
Polysyndactylism	11	0	9	0
Brachydactylism	11	0	9	0
Syndactylism	11	0	9	0
Digit Integrity	11 (yes)	0 (no)	7 (yes)	2 (no)

X-ray screen

$Chd7^{tm2a}$

Dysmorphology	normal $Chd7^{+/+}$	abnormal $Chd7^{+/+}$	normal $Chd7^{+/tm2a}$	abnormal $Chd7^{+/tm2a}$
Skull shape	5	0	5	1
Zygomatic bone	5	1	5	0
Maxilla	5	1	5	0
Scapula	6	0	6	0
Clavicle	6	0	6	0
Normal Number of Ribs (Right)	6	0	4	2 (2 with 1 missing rib)
Normal Number of Ribs (Left)	5	1 (1 with 1 missing rib)	5	1 (1 with 1 missing rib)
Shape of ribs	5	1	5	1
Shape of spine	2	4	1	5
Scoliosis	5	1	5	1
Normal Number of cervical vertebrae	6	0	6	0
Humerus	6	0	6	0
Radius	6	0	6	0
Ulna	6	0	6	0
Femur	6	0	6	0
Tibia	6	0	6	0
Shape of Ribcage	6	0	6	0
Fusion of Ribs	6	0	6	0
Pelvis	5	1	6	0
Normal number of Thoracic vertebrae	6	0	5	1 (1 with 1 missing)
Normal number of Lumbar vertebrae	6	0	4	2 (2 with 1 extra)
Normal number of Pelvic vertebrae	4	2 (2 with 1 extra)	1	(5 with 1 extra)
Normal number of Caudal vertebrae	6	0	6	0
Transition vertebrae	6	0	6	0
Shape of vertebrae	5	1	5	1
Fusion of vertebrae	2 (yes)	4 (no)	3 (yes)	3 (no)
Processes on vertebrae	6	0	5	1
Transverse Processes	6	0	5	1
Fusion Processes	6	0	4	0
Fibula	6	0	6	0
Normal number of digits	6	0	6	0
Polysyndactylism	6	0	6	0
Brachydactylism	6	0	6	0
Syndactylism	6	0	6	0
Digit Integrity	6 (yes)	0 (no)	6 (yes)	0 (no)

Supplementary Table S5: Plasma chemistry in 7 animals of each genotype for *Chd7*$^{+/tm2a}$ and 12 wild-type and 11 mutants for the *Chd7*$^{+/Whi}$ model. * p<0.05, ** p<0.005, *** p<0.0005. NA not available.

Plasma chemistry

	n= 7 wt vs n= 7 *Chd7*$^{+/tm2a}$	n= 11 wt vs n= 9 *Chd7*$^{+/Whi}$
Sodium (mM)	143.8 ± 6.2 vs 141.7 ± 1.1 ; ns	147.7 ± 0.7 vs 146.4 ± 1 ; ns
Potassium (mM)	4.3 ± 1.2 vs 4.2 ± 0.2 ; ns	4.8 ± 0.2 vs 4.1 ± 0.2 ; *
Chloride (mM)	107 ± 3.2 vs 105.6 ± 0.5 ; ns	108.7 ± 0.5 vs 111 ± 1.1 ; ns
Glucose (mM)	24.1 ± 13.8 vs 26.7 ± 1.3 ; ns	23.1 ± 2.1 vs 27.7 ± 2.5 ; ns
Fructosoamine	NA	202.5 ± 4 vs 191.3 ± 5.2 ; ns
Triglycerides (mM)	1.4 ± 1.3 vs 1.7 ± 0.2 ; ns	3 ± 0.2 vs 1.6 ± 0.4 ; **
Cholesterol (mM)	6.6 ± 3.2 vs 5.7 ± 0.4 ; ns	6.2 ± 0.1 vs 5 ± 0.3 ; **
High Density Lipoprotein (mM)	4.6 ± 1.8 vs 4.2 ± 0.3 ; ns	4.6 ± 0.1 vs 3.9 ± 0.2 ; **
Low Density Lipoprotein (mM)	1.5 ± 0.7 vs 1.1 ± 0.1 ; *	1 ± 0 vs 0.8 ± 0.1 ; **
Non-Esterified Free Fatty Acids (mM)	0.5 ± 0.5 vs 0.6 ± 0 ; ns	0.9 ± 0 vs 0.6 ± 0.1 ; ***
Glycerol (uM)	228.3 ± 101.2 vs 233.1 ± 25.1 ; ns	312.7 ± 22.5 vs 226 ± 24.3 ; *
Amylase (U/l)	1115.6 ± 381.7 vs 986.8 ± 34.2 ; ns	841.1 ± 25.5 vs 736.3 ± 50.2 ; ns
Alanine Aminotransferase (U/l)	96.5 ± 118.2 vs 47.8 ± 10.7 ; *	88.5 ± 9.5 vs 57.4 ± 12 ; ns
Alkaline Phosphatase (U/l)	83.5 ± 31.6 vs 113.1 ± 8.3 ; *	99.8 ± 5.8 vs 89.2 ± 4.7 ; ns
Creatine Kinase (U/l)	326.9 ± 435.2 vs 312.6 ± 86.7 ; ns	97.1 ± 23.6 vs 168.9 ± 63.3 ; ns
Aspartate Aminotransferase (U/l)	103.1 ± 90.2 vs 70.7 ± 7.5 ; ns	110.6 ± 11.8 vs 65 ± 10.6 ; *
Total Bilirubin (µM)	2.6 ± 1.5 vs 2.5 ± 0.4 ; ns	2.6 ± 0.1 vs 2.5 ± 0.2 ; ns
Total Protein (g/l)	50.7 ± 7.9 vs 46.9 ± 0.7 ; *	55.4 ± 0.8 vs 48.8 ± 1.6 ; **
Albumin (g/l)	24.6 ± 6.4 vs 22.6 ± 0.4 ; ns	28.3 ± 0.4 vs 24.5 ± 0.9 ; **
Creatinine (uM)	8.3 ± 2.2 vs 8.4 ± 0.5 ; ns	7 ± 0.3 vs 6.7 ± 0.3 ; ns
Urea (mM)	8.4 ± 3.2 vs 9.6 ± 0.5 ; ns	7.9 ± 0.2 vs 8.6 ± 0.1 ; *
Calcium (mM)	2.38 ± 0.3 vs 2.32 ± 0.03 ; ns	2.47 ± 0.02 vs 2.28 ± 0.03 ; ***
Magnesium (mM)	0.73 ± 0.2 vs 0.76 ± 0.03 ; ns	0.73 ± 0.01 vs 0.67 ± 0.02 ; *
Iron (µM)	37.6 ± 13.6 vs 30.6 ± 2.7 ; ns	39.5 ± 1.4 vs 38.4 ± 1.1 ; ns
Phosphate (mM)	3 ± 0.9 vs 2.7 ± 0.1 ; ns	2.9 ± 0.2 vs 3.4 ± 0.1 ; ns
Lactate Dehydrogenase (U/l)	759.6 ± 582.5 vs 599.9 ± 46.8 ; ns	567 ± 25.8 vs 466.8 ± 59.8 ; ns
Uric Acid (uM)	17.7 ± 17.2 vs 41.4 ± 10 ; *	24 ± 2.7 vs 25.7 ± 2.5 ; ns

Supplementary Table S6: Haematology components in 7 animals of each genotype for *Chd7*$^{+/tm2a}$ and 12 wild-type and 11 mutants for the *Chd7*$^{+/Whi}$ model. * p<0.05 using a t-test.

Haematology terminal

	n= 7 wt vs n= 7 Chd7$^{+/tm2a}$	*n= 11 wt vs n= 9 Chd7*$^{+/Whi}$
White Blood Cell Count (10^3/ul)	8.5 ± 4.4 vs 6.1 ± 0.7 ; *	7.2 ± 3.7 vs 6.6 ± 0.6 ; ns
Red Blood Cell Count (10^6/ul)	10.9 ± 0.9 vs 9.6 ± 0.6 ; ns	9.4 ± 2 vs 9.1 ± 0.2 ; ns
Mean Corpuscular Volume (fl)	54.4 ± 4.8 vs 54.7 ± 0.6 ; ns	52.8 ± 11.1 vs 50.6 ± 1.3 ; ns
Haemoglobin (g/dl)	16.9 ± 1.4 vs 15.1 ± 0.8 ; *	14.6 ± 3.4 vs 13.8 ± 0.3 ; ns
Haematocrit (%)	59.4 ± 3.5 vs 52.4 ± 2.8 ; *	49.8 ± 13.9 vs 45.9 ± 1.4 ; *
Mean Corpuscular Haemoglobin (pg)	15.5 ± 1.7 vs 15.8 ± 0.3 ; ns	15.5 ± 1.8 vs 15.2 ± 0.2 ; ns
Mean Corpuscular Haem. Conc. (g/dl)	28.5 ± 0.9 vs 28.9 ± 0.2 ; ns	29.4 ± 3.6 vs 30.2 ± 0.6 ; ns
Red Blood Cell Distribution Width (%)	11.9 ± 3.1 vs 12.3 ± 0.2 ; *	12.1 ± 2.4 vs 12.1 ± 0.2 ; ns
Platelet Count (10^3/ul)	1075 ± 470.5 vs 1033.1 ± 51.9 ; ns	1014.9 ± 448.4 vs 933.8 ± 47.6 ; ns
Mean Platelet Volume (fl)	5 ± 0.4 vs 5.1 ± 0.1 ; ns	6.1 ± 1.4 vs 6.2 ± 0.1 ; ns

Supplementary Table S7: Immunology and peripheral blood leukocytes expressed in % in 7 animals of each genotype for *Chd7*$^{+/tm2a}$ and 12 wild-type and 11 mutants for the *Chd7*$^{+/Whi}$ model. * $p<0.05$ using a t-test.

Immunology - Peripheral Blood Leukocytes Terminal

	n= 7 wt vs n= 7 Chd7$^{+/tm2a}$	*n= 11 wt vs n= 9 Chd7*$^{+/Whi}$
T cell CD3+ (%)	16.3 ± 18.1 vs 21.5 ± 2.5 ; ns	12.5 ± 12 vs 15 ± 2 ; ns
T cell CD4+ (%)	10.9 ± 14.8 vs 13.6 ± 1.6 ; ns	7.5 ± 7.6 vs 8.7 ± 1.5 ; ns
Treg cell CD25+ (%)	7 ± 7.6 vs 6.3 ± 0.9 ; ns	5.9 ± 3.4 vs 5.5 ± 0.4 ; ns
T cell CD8+ (%)	4.4 ± 4.5 vs 6.2 ± 0.8 ; ns	4.9 ± 6.4 vs 6 ± 0.8 ; ns
B cell CD19+ (%)	38.7 ± 36.9 vs 39.6 ± 2.6 ; ns	27.7 ± 15.8 vs 21.9 ± 3 ; ns
Mature B cell IgD+ (%)	96.2 ± 1.6 vs 96.1 ± 0.2 ; ns	93 ± 11.5 vs 92.4 ± 0.7 ; ns
Granulocyte Gr1+ (%)	27.4 ± 36.3 vs 19 ± 2.2 ; ns	42.3 ± 24.5 vs 47.3 ± 6 ; ns
Granulocyte Gr1+ (%)	27.4 ± 36.3 vs 19 ± 2.2 ; ns	Not done
NK cell (%)	2.1 ± 3.2 vs 3.5 ± 0.5 ; *	3 ± 2.8 vs 4.1 ± 0.8 ; ns
Monocyte (%)	7.7 ± 8.1 vs 5.8 ± 1.4 ; ns	8.7 ± 8.1 vs 3.7 ± 0.6 ; *

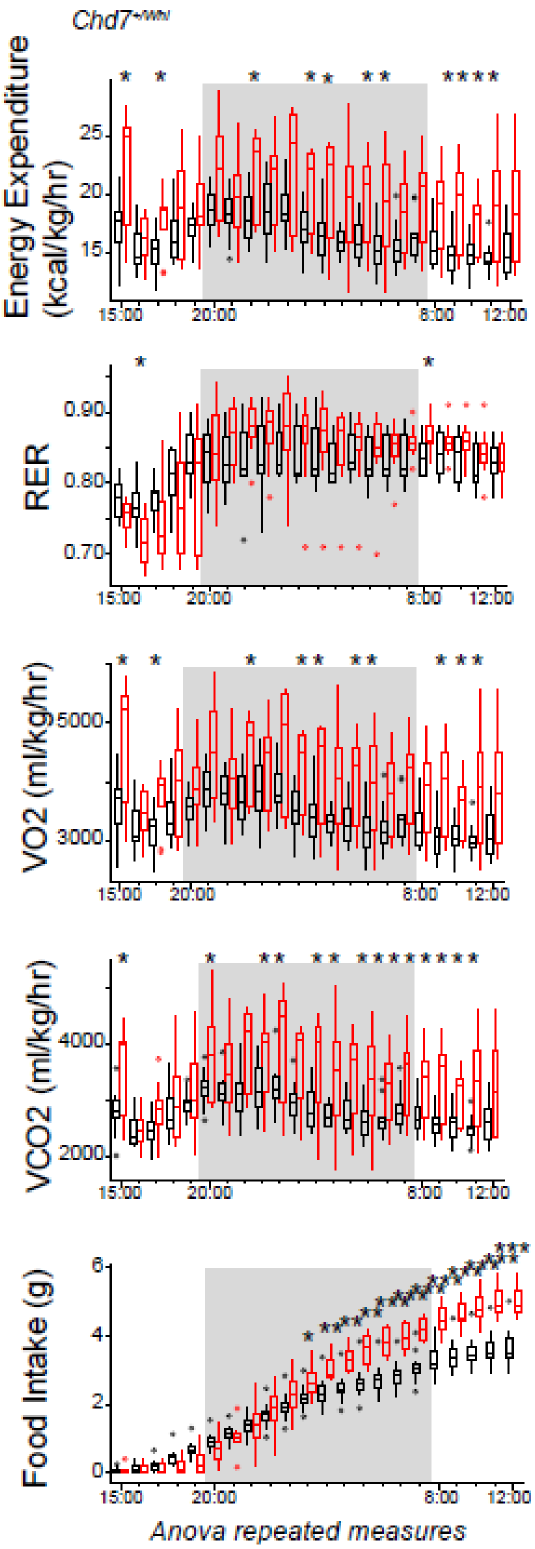

Supplementary Figure S1: Metabolic function in 12 week old *Chd7*$^{+/Whi}$ mice housed individually in calorimetry cages for a period of approximately 21 hours from approximately 2pm. From top to bottom: Energy expenditure, respiratory exchange ratio, volume of oxygen and carbon dioxide produced and food intake using box plots representing mean ± sem (boxes) with outliers (whiskers). $p < 0.05$, * $p < 0.005$, ** $p < 0.0005$, *** $p < 0.00005$.
